# Antineutrophil Cytoplasmic Antibody-Associated Glomerulonephritis in a Case of Scleroderma After Recent Diagnosis With COVID-19

**DOI:** 10.7759/cureus.12485

**Published:** 2021-01-04

**Authors:** Mojgan Jalalzadeh, Julio C Valencia-Manrique, Noella Boma, Ashok Chaudhari, Shobhana Chaudhari

**Affiliations:** 1 Internal Medicine/Nephrology, Metropolitan Hospital Center, New York Medical College, New York, USA; 2 Internal Medicine, Metropolitan Hospital Center, New York Medical College, New York, USA; 3 Internal Medicine/Geriatrics, Metropolitan Hospital Center, New York Medical College, New York, USA

**Keywords:** scleroderma, scleroderma renal crisis, anca-associated vasculitis, myeloperoxidase (mpo), kidney injury, covid-19

## Abstract

Antineutrophil cytoplasmic antibody (ANCA)-associated vasculitis (AAV) is a rare occurrence in systemic sclerosis (SSc) patients. AAV is an inflammatory disease that can lead to kidney failure due to the infiltration of mononuclear cells and the destruction of blood vessels. Also, crescentic glomerulonephritis (GN) has rarely been reported with coronavirus disease 2019 (COVID-19) and acute tubular injury is the most common renal pathology lesion in these patients. We present a rare case of a 46-year-old woman with SSc with new onset of renal failure after a recent diagnosis of COVID-19. Her serology was positive for p-ANCA and myeloperoxidase antibodies. Kidney biopsy was done and showed crescentic GN.

We suggest during this pandemic, patients with an immunological disorder that are infected with COVID-19 be closely monitored for any organ involvement.

## Introduction

Systemic sclerosis (SSc) is a multisystem disease characterized by fibrosis of the skin and internal organs, autoimmunity production and small vessel vasculopathy [1]. Clinically SSc classified into two subsets based on the extent of skin thickening. In patients with limited cutaneous SSc, thickening of the skin is limited on the face, hands, and arms, while in patients with diffuse cutaneous SSC, thickening of the skin affects the chest, abdomen, and/or upper arms [1].

SSc has a wide spectrum of clinical manifestations according to the organ which involved; organ involvement including skin, gastrointestinal, pulmonary, cardiac, renal, musculoskeletal, and less common neurologic abnormalities [2].

Laboratory tests to support SSc include positive anti-topoisomerase I (anti-Scl-70) antibody, anti-centromere antibody, and/or anti-ribonucleic acid (RNA) polymerase III antibody or anti-nuclear positive (ANA) antibody with nucleolar pattern. Anti-nuclear antibodies may be present in more than 90% of cases of systemic sclerosis, and at least one of the more specific antibodies (anti-centromere, anti-Scl-70, and anti-RNA polymerase III) is present in up to 70% of cases [2].

Clinical kidney disease is seen in half of patients. Scleroderma renal crisis (SRC) is the most serious kidney problem that occurs in 10 to 15% of the patients with diffuse cutaneous SSC and only 1-2% in the patients with limited cutaneous SSc [3]. Renal manifestation in SRC is edematous stenosis or destruction of interlobular arteries and arcuate arteries because of endothelial cell injury [4]. Anti-RNA-polymerase III antibodies have been found to be associated with an increased risk for SRC [5]. Clinically, patients with SRC present with new-onset hypertension, hypertensive retinopathy, seizures, pulmonary edema, microangiopathic hemolytic anemia, thrombocytopenia, elevated creatinine, mild hematuria, and proteinuria. Treatment for SRC includes use of angiotensin-converting enzyme (ACE) inhibitors [6].

Antineutrophil cytoplasmic antibody (ANCA)-associated glomerulonephritis (AAV) is rarely reported with SSc, in around 2.5% to 9% of patients [7]. Systemic manifestations often include pulmonary and/or renal involvement. Treatment of AAV is different from SRC and usually includes immunosuppressants and dialysis, if needed [8].

Here we report a case of a 46-year-old woman with SSc and recent diagnosis of COVID-19 who had new onset of kidney injury and clinical symptoms suggesting SRC but was found to have pauci-immune (P-ANCA) glomerulonephritis.

## Case presentation

This is a 46-year-old white Hispanic female with type-2 diabetes mellitus and scleroderma, who presents to the emergency room complaining of worsening dyspnea for the past two days associated with two months of epigastric pain, intermittent vomiting, diarrhea, poor appetite, and abdominal bloating. For four months weakness, significant weight loss of 10 kg, on and off cough with trace of epistaxis and hemoptysis.

She was diagnosed for SSc in 2005, complicated by pericarditis. Of note, she was recently (six months ago, 4/2020) diagnosed with COVID-19 with self-quarantine. The patient’s medication includes metformin (off for several months) and omeprazole.

On physical examination, she was hypertensive to 153/83 mmHg, had bilateral lower extremity edema, and skin thickening of the fingers of both hands extending proximal to the metacarpophalangeal joints, skin thickening and telangiectasia of the face, consistent with SSc, hypertensive retinopathy, and respiratory distress.

**Figure 1:**
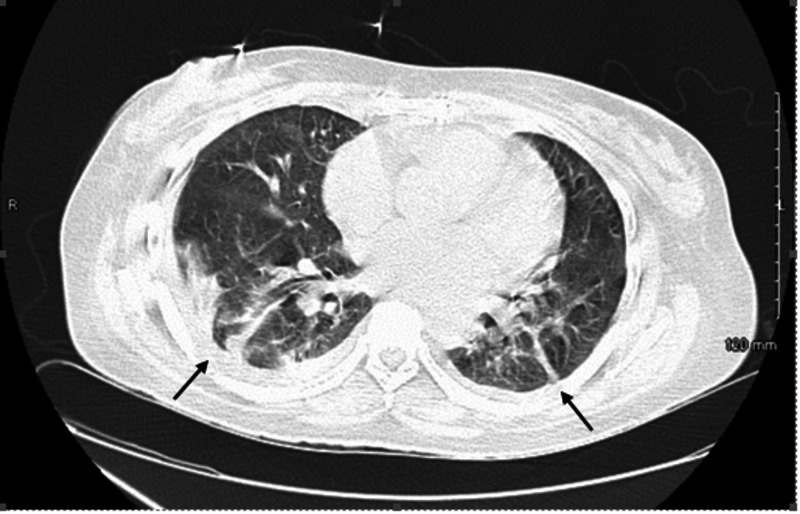
CT chest (axial lung window) showed bilateral pleural effusions and pulmonary infiltrates

Her laboratories were noted elevated blood urea nitrogen 84 mg/dL and creatinine 8.3 mg/dL. Baseline value was 0.4 mg/dL almost a year ago. Urinalysis revealed active sediment (large blood, red blood cell (RBC) 100/HPF, white blood cell (WBC) 0-2/HPF, few epithelial cells, with no casts), and non-nephrotic range proteinuria (urine albumin-creatine ratio 1608 mg/g, six months ago was 38 mg/g). Her complete blood count was significant for the decreased hemoglobin of 5.4 g/dl and platelet of 80,000/mcl. Echocardiography (ECG) was normal with no evidence of pulmonary hypertension. CT scan of chest, Figure 1, showed bilateral pleural effusions and pulmonary infiltrates. Ultrasound of the kidney and bladder showed echogenic kidneys with lower normal size (right 8.7 cm, left 9.2 cm) with no evidence of obstruction.

Because of flash pulmonary edema, anemia, thrombocytopenia, non-oliguric acute kidney injury (AKI), and new onset of hypertension with hypertensive retinopathy, she was started on captopril for concern of SRC. Stool was positive for *Escherichia coli* 0157 and enterotoxigenic *E. coli.* However, thrombotic thrombocytopenic purpura (TTP) and hemolytic uremic syndrome (HUS) were ruled out with normal ADAMTS13 (a disintegrin and metalloproteinase with a thrombospondin type 1 motif, member 13) results and negative markers of hemolysis (no schistocytes, bilirubin T 0.3 mg/dL, bilirubin D <0.1mg/dL, lactate dehydrogenase (LDH) 193 u/l, haptoglobin 173 mg/dL), respectively.

Her serologies were positive for anti-ribonuclear protein >8, ANA at 1/2560, chromatin Ab at 6.6, P-ANCA >1/1280, and myeloperoxidase Ab at a level of 161.8 unit. C-ANCA and Proteinase 3 Ab were negative. Inflammatory markers were elevated: erythrocyte sedimentation rate (ESR) 85 mm/h, C-reactive protein (CRP) 9.1 mg/dL. Other relevant serology and biochemistry (RNA polymerase III IgG, anti-Smith Ab, centromere Ab, double-stranded DNA (dsDNA) Ab, cardiolipin Ab, glomerular basement membrane (GBM) Ab, cryoglobulins, complement, virology) were normal. COVID-19 Ab was positive, IgG (1.68 S/C, reference range <1.39 S/C), and polymerase chain reaction (PCR) test was negative.

**Figure 2:**
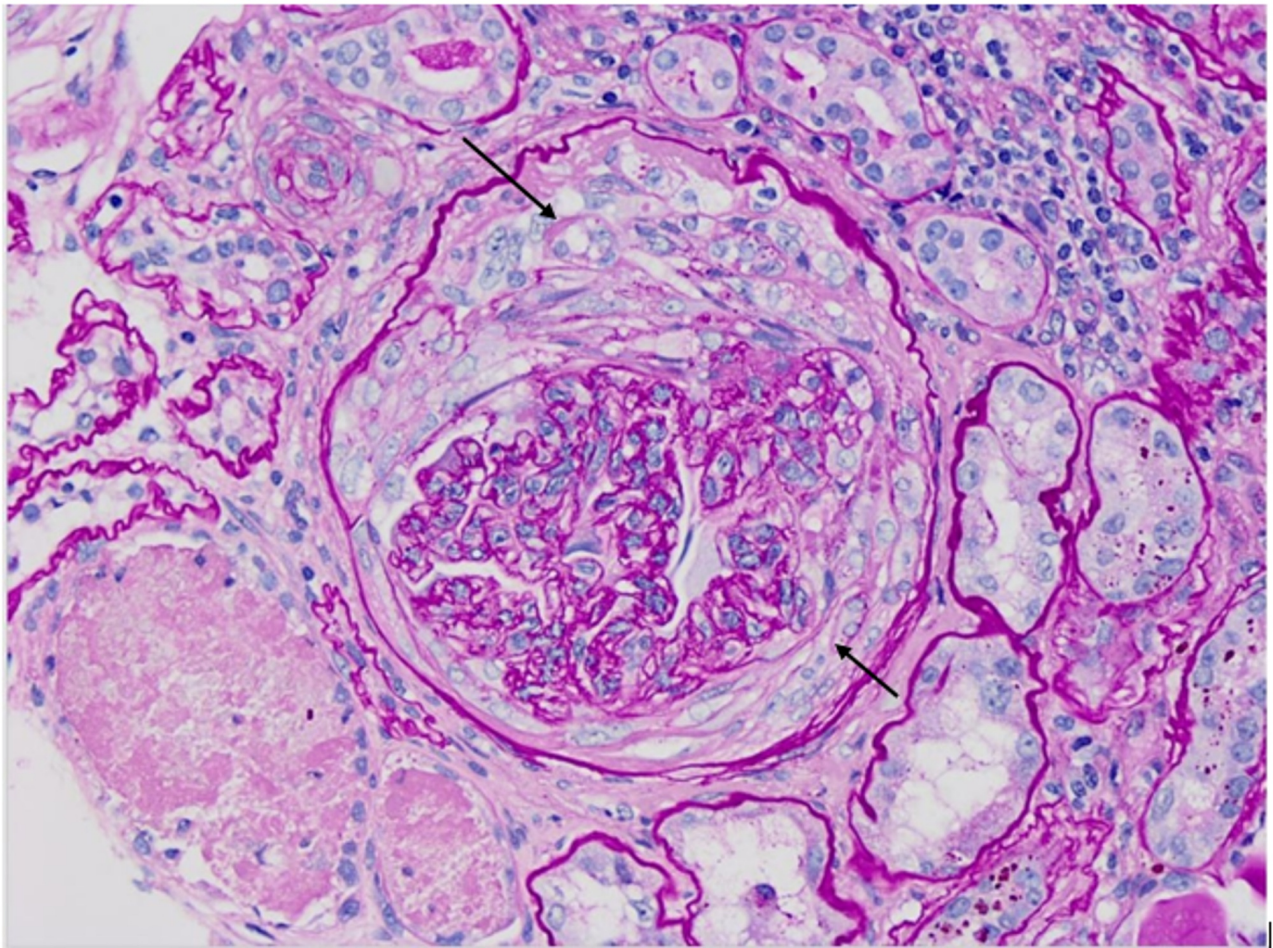
Fibro-cellular crescent (Periodic acid–Schiff staining, 40x)

**Figure 3:**
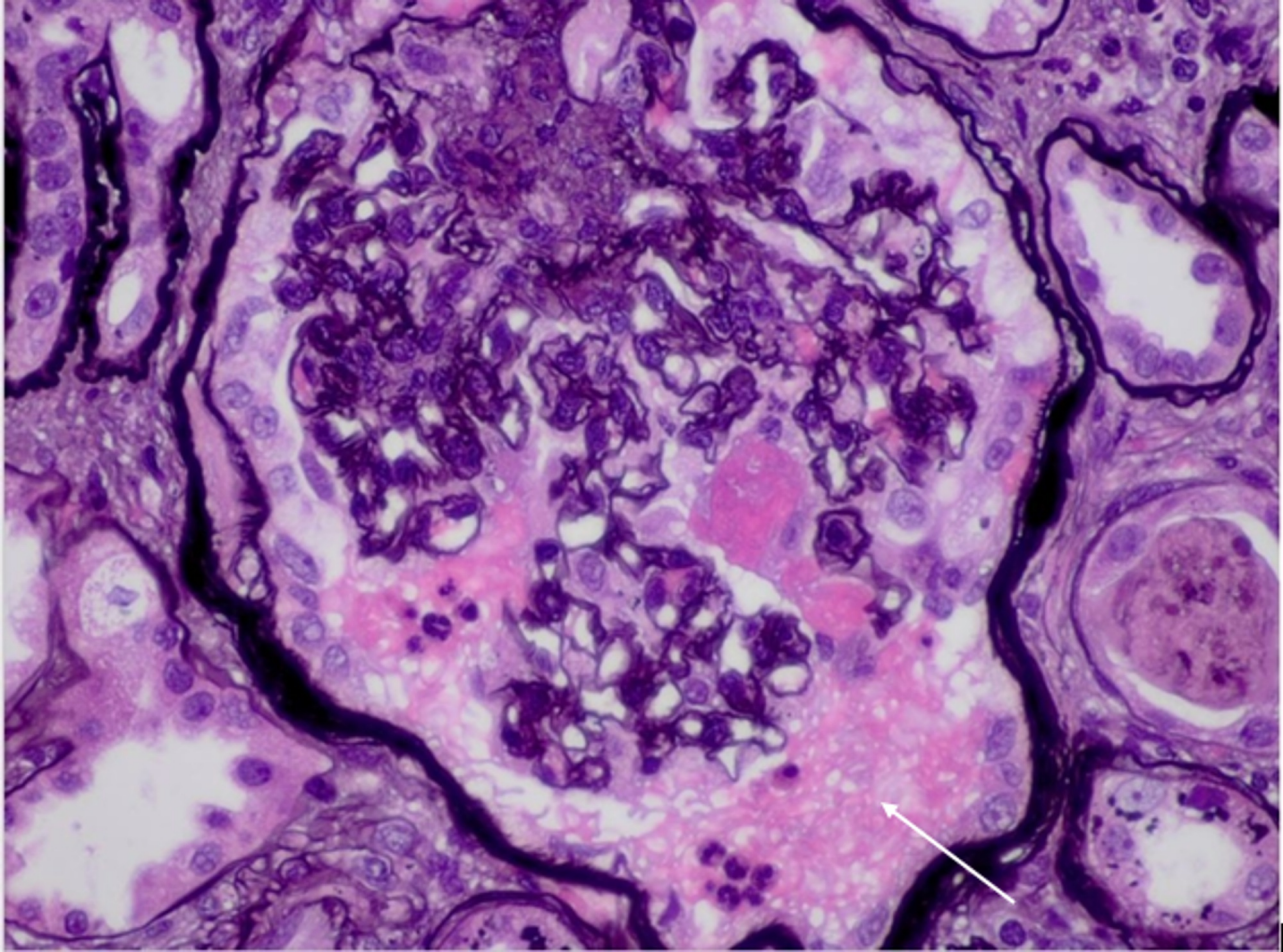
Fibrinoid changes (Jones staining, 60x)

**Figure 4:**
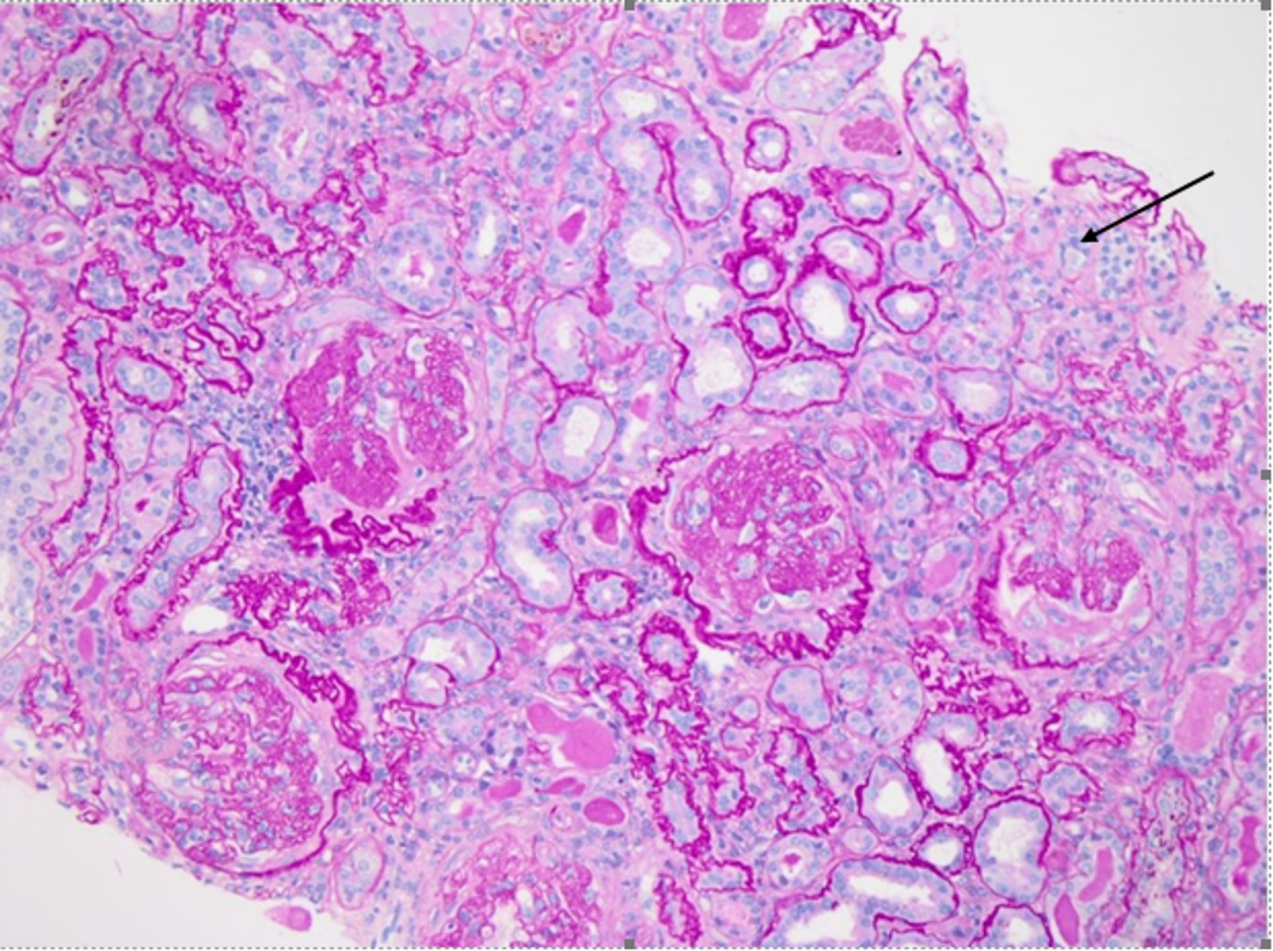
Fibrosis and tubular atrophy (Periodic acid–Schiff staining, 20x)

Patient was started intravenous methylprednisolone 1000 mg for three doses for suspicion of ANCA vasculitis leading to nephropathy. Kidney biopsy was consistent with a crescentic glomerulonephritis (GN), as demonstrated in the light microscopic of Figures 2, 3, 4.

The figures show the following:

1. Chronic sclerosing glomerulonephritis (GN) with focal crescents, pauci-immune (P-ANCA associated). Forty-five of 48 glomeruli are globally sclerotic or approaching global sclerosis. A subset of these globally sclerotic glomeruli demonstrates dissection of the glomerular tuft by fibrosis and rupture of the Bowman’s capsule, suspicious for old fibrous crescents.

2. Tubular atrophy and severe interstitial fibrosis.

3. Mild arteriosclerosis.

Microscopic immunofluorescence results also support the diagnosis of pauci-immune GN.

Nephrologist’s plan is to start dialyzing in the future given 93% sclerosed glomeruli in biopsy results. Rheumatologist’s plan is induction with IV rituximab after pulmonary clearance since the result of QuantiferonTB was indeterminate.

## Discussion

The antineutrophil cytoplasmic antibody (ANCA) is a class of Immunoglobulins with subtypes of c-ANCA and p-ANCA, which are mainly produced against cytosolic antigens of proteinase 3 (PR3) and myeloperoxidase (MPO), respectively. These antibodies can develop small vessel vasculitis [9]. In SSc patients, ANCA-associated vasculitis is rare but has serious consequences.

Arad et al. [10] in 2011 reported three patients with diffuse form of SSc and AAV, and that SRC typically occurs at the early period of SSc, while AAV occurs after several years of illness. In a cohort study using a research database on 2,200 SSc patients between 1999 and 2010, the researcher obtained useful information on nine of 35 patients who fitted the criteria for SSc and vasculitis. Among them, seven patients had diffuse form of SSc and one patient had the localized form. MPO-ANCA was positive in seven patients and anti-proteinase-3 in one patient [11].

P-ANCA vasculitis occurs most commonly in women with limited scleroderma or CREST, as well as those with Sicca syndrome or overlap connective tissue disorder features. Severe manifestations including pulmonary/renal syndrome and death may occur, and treatment with high-dose corticosteroids and cyclophosphamide seems to have benefits [12].

Here, we present a case of SSc that was diagnosed 10 years ago without significant organ involvement and was not on any immunosuppressive medications. After involvement with COVID-19, she started to feel weakness, fatigue, loss of appetite, unintended weight loss, and the rest of signs and symptoms that previously mentioned. She did not seek any medical help since she thought all changes were related to COVID-19 and would improve by themselves over time. At time of presentation, it was thought patient has AKI due to SRC, but later kidney biopsy showed that she has AAV. More than 93% of glomeruli were sclerosed in biopsy and patient planned for maintenance renal replacement therapy.

COVID-19 is a new viral pandemic which has dramatically driven medical attention. The most common report of COVID-19 and kidney involvement is AKI, mainly due to acute tubular necrosis [13]. Cases of thrombotic microangiopathy and collapsing glomerulopathy with COVID-19 have been reported [14,15].There is some hypothesis that the virus can directly invade endothelial cells, causing vasculitis [16,17], and depending on the vessel that is affected, can present with different forms of vasculitis [18].

To date, only three cases of AAV with COVID-19 have been reported, one in Iran and two in the United States [19,20]. We suspect that our patient developed GN during or after her COVID-19 infection, and gradually other manifestations of kidney dysfunction developed.

## Conclusions

Clinicians should be aware of AAV with GN as another potential pathology in patients of SSc with renal involvement. Kidney biopsy should be performed to ensure the best care. Also, in the era of the COVID-19 pandemic coexistence of glomerulonephritis with COVID-19 should be considered. We recommend during this pandemic, patients with immunologic disorders that are infected with COVID-19 be evaluated more frequently and closely monitored for any organ involvement.
